# 

**Published:** 1924-01

**Authors:** 


JANUARY, 1924.

*Vol. XLI.* *No.* **151.**

### THE BRISTOL MEDICO-CHIRURGICAL JOURNAL

*PUBLISHED UNDER THE AUSPICES OF*

*THE BRISTOL MEDICO-CHIRURGICAL SOCIETY.*

Library of Congress MAR 14 1924 Smithsonian Deposit

*Editor:* P. WATSON-WILLIAMS, M.D.,

WITH WHOM ARE ASSOCIATED

JAMES SWAIN, C.B., C.B.E., M.S., M.D.,

J. LACY FIRTH, M.S., M.D., CAREY COOMBS, M.D.

J. A. NIXON, C.M.G., M.D., *Assistant Editor.*

*Editorial Secretary:* A. L. FLEMMING, M.B., B.Ch.

BRISTOL: J. W. ARROWSMITH LTD., 11 QUAY STREET.

LONDON: J. W. ARROWSMITH (LONDON) LTD., 6 UPPER BEDFORD PLACE, RUSSELL SQUARE.

*Price Three Shillings net.*

PRINTED IN GREAT BRITAIN.

